# Improving the Documentation of Clinical Frailty and Resuscitation Status on a Psychiatric Older Adult Ward: A Quality Improvement Project

**DOI:** 10.7759/cureus.115979

**Published:** 2026-09-09

**Authors:** Christianna Ramsundar, Oliver Tran, Ekaterina Doukova, Galina Nikolova

**Affiliations:** 1 Psychiatry, North London NHS Foundation Trust, London, GBR

**Keywords:** clinical frailty, dnacpr, inpatient psychiatry services, psychiatry of old age, quality improvement projects

## Abstract

Background

Older adults admitted to psychiatric inpatient wards frequently have significant physical health comorbidities, placing them at elevated risk of deterioration and death. Despite national guidance, documentation of clinical frailty assessments and do not attempt cardiopulmonary resuscitation (DNACPR) decisions in this setting remains inconsistent. This project was therefore implemented to improve the documentation of resuscitation decisions and frailty assessments on The Oaks, which is a 20-bedded acute psychiatric admissions ward for adults over 65 years at Chase Farm Hospital, North London NHS Foundation Trust.

Method

A quality improvement project (QIP) was conducted across four phases between August 2024 and April 2025. Baseline data collection (Phase 1) examined 20 current inpatients for documentation of frailty assessment using the Rockwood Clinical Frailty Scale (RCFS) and DNACPR status in the electronic patient record (RiO). Following multidisciplinary team (MDT) discussion (Phase 2), a standardised pathway was implemented which included routine frailty assessment on admission, MDT safety huddle discussion, patient and family information provision (Phase 3, including a bespoke CPR/DNAR information leaflet), and completion of DNACPR forms at care planning meetings. Re-evaluation (Phase 4) examined notes for all patients admitted over a one-month period (21 January to 21 February 2025).

Results

Following implementation of the intervention, DNACPR form completion increased from 5% to 30% (absolute increase: 25 percentage points; Fisher's exact test: p = 0.095). Documentation of DNACPR discussions increased from 15% to 90% (absolute increase: 75 percentage points; Fisher's exact test: p < 0.001), representing a sixfold relative increase. Where forms were not completed, reasons were clearly documented. Of the two project objectives, DNACPR documentation was measured as the primary re-evaluation outcome. Frailty documentation was not formally re-evaluated; however, this will be assessed in future QI cycles to accurately reflect the study findings.

Conclusion

This QIP demonstrates that a structured, MDT-driven pathway can produce short-term feasibility and a substantial improvement in DNACPR discussion documentation on a single older adult psychiatric ward. The pathway is low cost and has been adopted as standard practice, with ongoing monitoring planned. Further cycles targeting frailty scoring completion and DNACPR form rates are planned.

## Introduction

Problem 

The Oaks is an acute inpatient psychiatric ward and functions to assess and treat older adults with both functional and organic psychiatric disorders. It is located within the North London NHS Foundation Trust and serves a wide catchment area, including the boroughs of Barnet, Enfield, Haringey, Camden and Islington. At the time of the project, the ward served areas of Barnet, Enfield and Haringey prior to a trust merger. Patients admitted to The Oaks are frequently medically complex and, in addition to their psychiatric diagnoses, possess multiple comorbidities.

A proportion of these patients are significantly frail. Frailty assessment allows clinical teams to stratify risk, identify patients at highest risk of deterioration, and tailor care plans accordingly, including decisions about the appropriate ceiling of treatment. Similarly, completing do not attempt cardiopulmonary resuscitation (DNACPR) decisions in advance is important because it prevents harmful or futile intervention in the event of cardiac arrest, reduces distress for patients and families during a crisis, and ensures care aligns with the patient's values and wishes. Full CPR is often not in the best interest of significantly frail patients, as it is unlikely to be successful and may cause additional harm and distress for both patients and relatives.

Prior to this quality improvement project (QIP), there was no organised process for assessing and documenting clinical frailty, nor was there a pathway for recording DNACPR discussions or the decision. The project had a dual aim: (1) to improve documentation of frailty assessments using the Rockwood Clinical Frailty Scale (RCFS) to 100% of eligible inpatients and (2) to improve documentation of DNACPR discussions and decisions to 100% of eligible inpatients both within 12 weeks of implementing the new pathway. Given the very low baseline frailty documentation rate and the clinical priority of first establishing a structured resuscitation discussion process, DNACPR documentation was prioritised as the primary measurable outcome in the re-evaluation phase, with frailty documentation pre-specified as the primary outcome for subsequent QI cycles.

Background 

Individuals with severe mental illness (SMI) experience significantly poorer physical health [1] than the general population. Life expectancy in those with SMI is 15 to 20 years shorter, with excess mortality predominantly attributable to natural causes including cardiovascular disease, respiratory illness, and metabolic disorders [1]. This mortality gap is further compounded in older adults, who are at heightened risk of developing multiple concurrent physical health conditions requiring parallel management alongside their psychiatric care.

Frailty is a recognised clinical syndrome characterised by a reduced physiological reserve and increased vulnerability to stressors, resulting in adverse health outcomes including falls, hospitalisation, functional decline, and death [2]. Emerging evidence indicates that frailty is more prevalent in people with SMI than in age-matched controls in the general population [3,4]. A systematic review by Pearson et al. [4] confirmed this association across multiple frailty measures, emphasising the importance of proactive frailty identification in psychiatric settings. Crucially, a longitudinal cohort study demonstrated that frailty independently predicts mortality within five years of psychiatric admission in older adults, over and above age, gender, and multimorbidity [5], establishing frailty assessment as a clinically significant component of the admission process. 

The RCFS, developed by Rockwood et al. [6], is a validated, widely adopted nine-point clinical tool that allows rapid assessment of frailty based on clinical judgement, ranging from 1 (very fit) to 9 (terminally ill). It has been endorsed by the British Geriatrics Society for use in acute and inpatient settings and has demonstrated strong predictive validity for adverse clinical outcomes [6,7]. 

The Clinical Frailty Scale (CFS) score provides a structured, evidence-based foundation for DNACPR discussions. Patients scoring CFS 5 (mildly frail) or above are at significantly increased risk of poor outcomes from CPR, and scores of CFS 7 or above (severely frail to terminally ill) are associated with a very low likelihood of survival to hospital discharge following attempted resuscitation. Incorporating the CFS into the admission process therefore enables clinicians to identify patients for whom a DNACPR conversation is clinically appropriate and to ground that conversation in objective clinical data, rather than subjective impression alone. This supports a more consistent, equitable, and evidence-based approach to advance care planning across the ward.

The General Medical Council (GMC) provides extensive ethical and legal guidance on DNACPR decisions [8]. The GMC emphasises that DNACPR decisions are clinical decisions made in advance to guide management in the event of a patient's cardiorespiratory arrest and that they are not legally binding directives but clinical recommendations which must be made with due regard to the patient's best interests, their expressed wishes, and the views of those close to them. The GMC further requires that decisions are clearly documented, regularly reviewed, and communicated to all relevant members of the care team. National guidance from the British Medical Association, Resuscitation Council UK, and Royal College of Nursing [9] reinforces that DNACPR decisions must be made following clinician assessment, patient and family discussion wherever possible, and clear documentation in the clinical record. The Resuscitation Council UK quality standards for mental health inpatient care [10] explicitly require that resuscitation decisions are documented, communicated to the team, and regularly reviewed.

Despite this, published evidence from old age psychiatry settings indicates persistent deficiencies in both DNACPR documentation and frailty assessment. A QI audit conducted across two old age psychiatry wards in NHS Fife [11] demonstrated that completion and online upload of DNACPR documentation were significantly below expected standards at baseline, with marked improvements achieved through targeted educational and workflow interventions. A multisite audit of older-age psychiatric inpatient wards found that DNACPR status was discussed on admission in only 8% of cases and documented at the first MDT in only 10% [12]. A contemporaneous audit across four old age psychiatry wards in Aneurin Bevan University Health Board identified particular deficits in documented discussion with patients, with patient discussion present in fewer than half of DNACPR forms reviewed [13]. Taken together, these findings demonstrate that poor resuscitation documentation is a systemic problem in older adult psychiatric inpatient care nationally. This QIP was undertaken to address these evidence-based shortfalls in practice on The Oaks ward, to establish a sustainable, embedded pathway for frailty assessment and DNACPR documentation as routine components of inpatient care. 

## Materials and methods

Baseline measurement 

Initial data collection (Phase 1) focused on searching electronic patient records for all inpatients as a first review of practice. A structured data collection proforma was designed to record patient identifying details, whether a frailty assessment and DNACPR form had been completed since admission, and whether there was documentation of a resuscitation discussion. Eligibility for DNACPR discussion was determined by the MDT and considered appropriate for all patients with a CFS score of 5 or above, or those with significant physical health comorbidities irrespective of frailty score, unless the patient lacked capacity and no appropriate consultee was available. For data collection purposes, a DNACPR discussion was coded as documented if the electronic patient record contained a clinical entry in a ward round note, multidisciplinary team (MDT) entry, or dedicated advance care planning field explicitly recording that resuscitation status had been discussed with the patient and/or their family or carers. A DNACPR form was coded as completed if a formal DNACPR form was present and uploaded in the electronic record.

Of the 20 patients reviewed at baseline, none had a frailty assessment documented. Three patients (15%) had a documented resuscitation discussion; in each case, this had taken place on a medical ward prior to admission to The Oaks. No DNACPR forms were completed or uploaded in electronic records for any of the 20 patients. One patient had an external DNACPR form completed at another hospital, which was not completed at The Oaks. These findings are shown in Figure 1. It should be noted that the baseline cohort was a prevalence sample, all patients currently admitted at a single point in time, which included long-stay patients with established care plans. This differs structurally from the re-evaluation cohort (Phase 4), which was an incidence sample of new admissions. This difference in sampling frame is acknowledged as a limitation.

**Figure 1:**
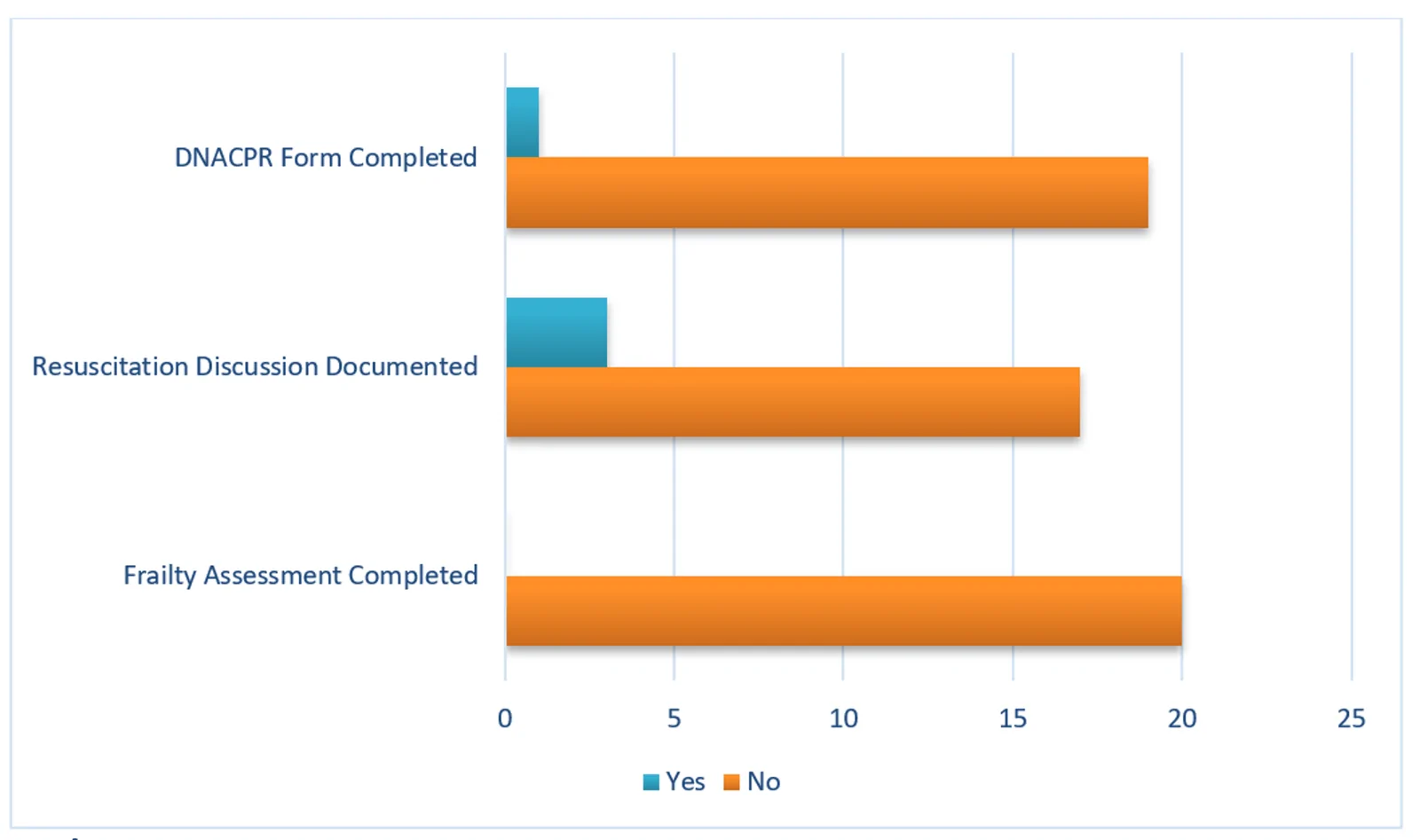
Baseline evaluation DNACPR: do not attempt cardiopulmonary resuscitation

Design 

This is a before-and-after QIP carried out on The Oaks, a 20-bedded older adult inpatient psychiatric ward. Baseline data were collected from all 20 inpatients on frailty assessment, resuscitation status, and DNACPR form completion. Findings were presented to the MDT, and a new pathway was developed collaboratively to improve documentation. The new pathway was implemented for all existing inpatients and all new admissions from October 2024 onwards. Whilst this project was not prospectively designed around formal Plan-Do-Study-Act (PDSA) cycles, the project phases are consistent with QI methodology and can be retrospectively mapped to a PDSA framework, as shown in Table 1 below. It is acknowledged that this mapping is retrospective and that formal iterative PDSA cycles were not conducted as part of the original project.

**Table 1:** Retrospective PDSA cycle mapping PDSA: Plan-Do-Study-Act; DNACPR: do not attempt cardiopulmonary resuscitation; MDT: multidisciplinary team; NS: not significant PDSA mapping is retrospective. The project was not prospectively designed around formal PDSA cycles

Phase	Plan	Do	Study	Act
Phase 1-2	Identify baseline documentation rates for frailty and DNACPR; MDT root cause analysis	Collect baseline data via RiO review; present findings at MDT safety huddle	0% frailty documentation; 15% DNACPR discussion; 5% form completion. Key barrier: no structured pathway	Agree five-step MDT-driven pathway; assign clinical ownership
Phase 3	Design patient/family information resources to support DNACPR discussions	Develop bespoke two-sided CPR/DNAR information leaflet	Families needed time to reflect; early conversations more effective than single-meeting approach	Initiate conversations early in admission rather than at care planning meeting alone
Phase 4	Re-evaluate DNACPR documentation rates post-implementation	Review RiO records for all admissions over one month (Jan-Feb 2025)	90% DNACPR discussion documented (p < 0.001); 30% form completion (p = 0.095, NS)	Embed pathway as standard practice; plan future cycle measuring frailty documentation

A root cause analysis identified several barriers to optimal documentation: the lack of a standardised admission process incorporating frailty assessment; the absence of a formal mechanism for identifying patients requiring DNACPR discussions; limited resources to support patient and family engagement; and no established stage in the clinical pathway at which DNACPR decisions were routinely discussed and documented.

A multicomponent pathway was designed with the MDT to address these barriers (Figure 2). The figure below illustrates the key elements of the pathway developed. The intervention was designed to be sustainable within existing ward structures, with no requirement for additional staffing or resources beyond the information leaflet.

**Figure 2:**
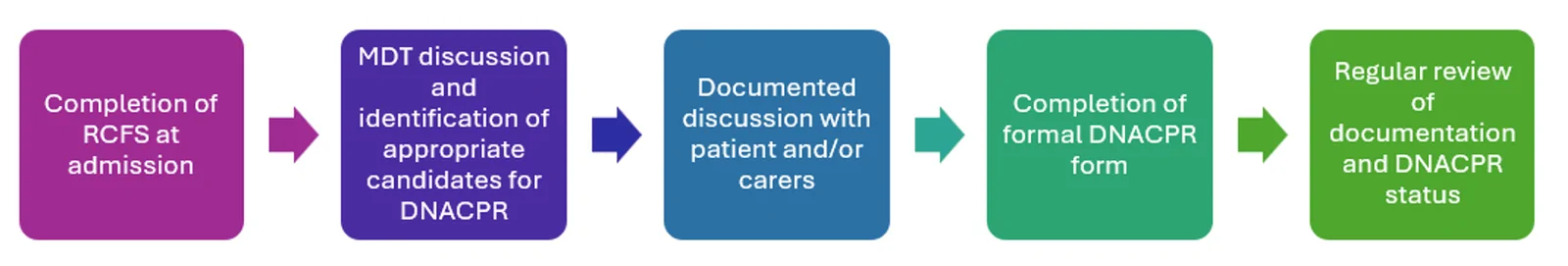
Multicomponent pathway elements RCFS: Rockwood Clinical Frailty Scale; MDT: multidisciplinary team; DNACPR: do not attempt cardiopulmonary resuscitation

The responsibility for completing the RCFS assessment rested with the admitting doctor as part of the standard clerking process, or the ward doctor within 72 hours of admission. The responsibility for initiating DNACPR discussions was shared across the MDT, with the consultant or their nominated deputy expected to lead initial conversations. Nursing staff and allied health professionals were involved in identifying patients ready for discussion and in providing the information leaflet to patients and families. The MDT safety huddle served as the forum for team-level review of each patient's frailty and resuscitation status. To support replication by other teams, the pathway steps are set out as an operational checklist in Table 2.

**Table 2:** Operational pathway checklist CFS: Clinical Frailty Scale; MDT: multidisciplinary team; DNACPR: do not attempt cardiopulmonary resuscitation

Step	Action	Responsible clinician	Timing	Documentation
1	Rockwood CFS assessment	Admitting doctor	Within 72 hrs of admission	RiO clinical entry
2	Review CFS score and identify patients for DNACPR discussion	Consultant/MDT	Next safety huddle	MDT huddle entry in RiO
3	Provide CPR/DNAR information leaflet to patient and/or family	Named nurse or admitting doctor	Within 72 hrs of admission	RiO nursing entry
4	DNACPR discussion with patient and/or family/carers	Consultant or nominated deputy	Within one week of admission	RiO clinical entry (ward round or dedicated ACP note)
5	Complete DNACPR form if clinically indicated and agreed	Consultant	At care planning meeting or following discussion	Formal DNACPR form uploaded to RiO

Strategy 

Phase 1: Baseline Review 

Baseline data were collected in August 2024, as described in the Measurement section. Findings were presented to the MDT to establish shared understanding of the current state of practice and to generate motivation for change. 

Phase 2: Pathway Design and Implementation 

The MDT discussed baseline findings at the ward safety huddle in October 2024. Key learning was that documentation gaps were not primarily attitudinal; clinicians understood the importance of DNACPR planning, but structural: there was no explicit prompt, no agreed ownership, and no defined point in the care pathway at which frailty assessment or DNACPR was expected to occur. Eleven patients were admitted to The Oaks during the re-evaluation period (21 January to 21 February 2025). One patient was excluded as they were transferred to another ward within a very short time of admission, before any clinical assessments could be completed. This left a final re-evaluation sample of 10 patients.

The five-step pathway was agreed collaboratively and implemented immediately. An initial practical challenge was the time required to gather comprehensive past medical history on newly admitted patients, particularly those admitted out of hours or in an agitated or confused state, before frailty could be meaningfully assessed. The team agreed that frailty scoring should be completed within 72 hours of admission wherever possible.

Phase 3: CPR/DNAR Patient Information Leaflet 

A bespoke two-sided CPR/DNAR information leaflet was designed for patients and relatives, provided in the appendix. This was developed following review of existing information materials from BEH NHS Mental Health Trust and other national organisations. The leaflet was written in accessible language and covered what CPR involves, what a DNACPR decision means, who makes the decision, how family members can be involved and what happens if a patient has no DNACPR form. The leaflet was introduced on the ward for use during admission discussions with patients and families. 

Phase 4: Re-evaluation 

Eleven patients were admitted to The Oaks between 21 January and 21 February 2025. One patient was excluded as they were transferred to another ward within a very short time of admission, before any clinical assessments could be completed. This left a final re-evaluation sample of 10 patients. The re-evaluation cohort comprised new admissions over a one-month incidence window, which differs structurally from the prevalence-based baseline cohort (all 20 patients currently admitted at a point in time). This change in sampling frame is acknowledged as a limitation in the discussion.

Statistical analysis

Fisher's exact test was used to compare proportions between the baseline and post-intervention periods, given the small sample sizes and expected cell frequencies below five. All tests were two-tailed, and statistical significance was defined as p < 0.05. Analysis was performed using GraphPad Prism version 11 (Released 2026; GraphPad Software, LLC; Boston, MA, USA).

## Results

DNACPR discussions were documented for nine of the 10 eligible patients (90%), compared with three of 20 (15%) at baseline. In the one case where a discussion was not documented, the clinical notes clearly recorded that a DNACPR discussion was not felt appropriate given minimal frailty and absence of significant physical health comorbidities, reflecting clinical judgement rather than a process omission, as shown in Figure 3 and in Table 7 in the appendix.

**Figure 3:**
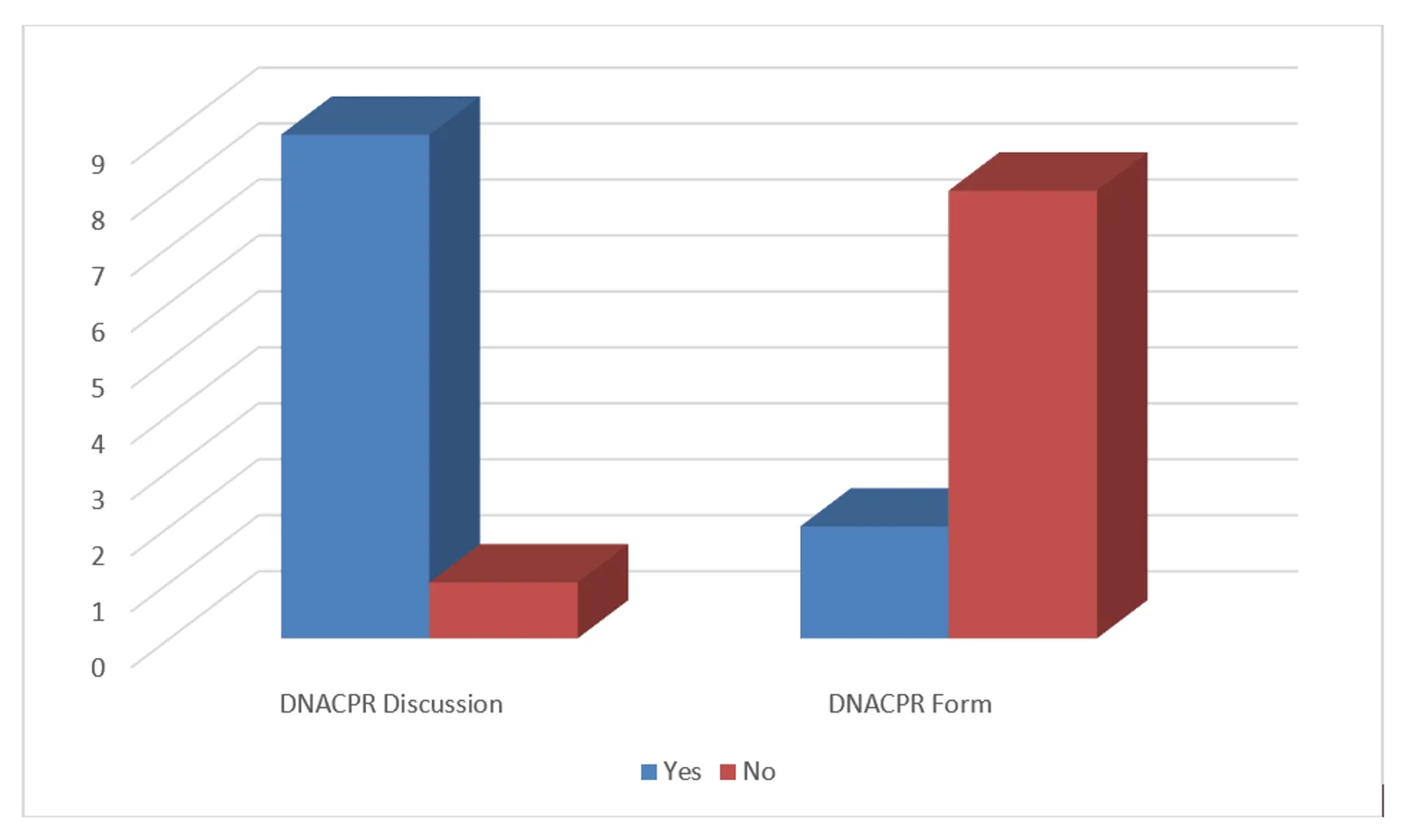
Post-intervention data DNACPR: do not attempt cardiopulmonary resuscitation

DNACPR forms were completed for three of the 10 patients (30%). In the remaining seven cases where forms were not completed, reasons were documented in the clinical notes. These were patient and/or family preference to remain for active CPR (two patients), transfer to another ward prior to completion of follow-up discussions (two patients), admission to a general medical ward prior to form completion (one patient), and significant clinical improvement rendering further DNACPR discussion no longer appropriate (one patient). These reasons reflected legitimate clinical and patient-centred considerations rather than process failures. 

Statistical analysis was performed using Fisher's exact test to compare proportions between the pre-intervention and post-intervention cohorts owing to small sample sizes. Statistical significance was defined as p < 0.05. Contingency 2 x 2 tables for DNACPR discussion and DNACPR form completion are shown in Tables 3-4, respectively.

**Table 3:** DNACPR discussion documented DNACPR: do not attempt cardiopulmonary resuscitation Fisher's exact test. p < 0.05 is considered statistically significant

	Yes	No	p-value
Baseline	3	17	
Post-intervention	9	1	
			<0.001

**Table 4:** DNACPR form completion DNACPR: do not attempt cardiopulmonary resuscitation Fisher's exact test. p < 0.05 is considered statistically significant

	Yes	No	p-value
Baseline	1	19	
Post-intervention	3	7	
			0.095

Following the implementation of the intervention, DNACPR form completion increased from 5% to 30% (absolute increase: 25 percentage points; Fisher's exact test: p = 0.095). Documentation of DNACPR discussions increased from 15% to 90% (absolute increase: 75 percentage points; Fisher's exact test: p < 0.001), representing a sixfold relative increase, as shown at baseline and post-intervention in Table 5. The improvement in DNACPR form completion rate (5% to 30%) did not reach statistical significance (Fisher's exact test: p = 0.095), likely reflecting insufficient statistical power given the small sample sizes. This finding should be interpreted cautiously and reassessed in future QI cycles with larger cohorts.

**Table 5:** Baseline and post-intervention statistics DNACPR: do not attempt cardiopulmonary resuscitation Statistical comparisons performed using Fisher's exact test. p < 0.05 considered statistically significant

Outcome	Baseline n/N (%)	Post-intervention n/N (%)	Absolute change	Relative risk	p-value
DNACPR form completion	1/20 (5%)	3/10 (30%)	+25 percentage points	6.0	0.095
DNACPR discussion documented	3/20 (15%)	9/10 (90%)	+75 percentage points	6.0	<0.001

Measurement of frailty assessment documentation was a co-primary objective of the project and is pre-specified as a primary outcome for future QI cycles. The focus of this re-evaluation was on DNACPR documentation as the primary endpoint given the clinical prioritisation described in the Problem section. The Rockwood scoring pathway was agreed as a standard part of the new admissions process, and frailty assessment documentation will form a formally measured primary outcome in the next QIP cycle. 

Data were collected from the electronic records by the project leads. No missing data were identified in Phase 4, and all included patients had sufficient clinical documentation to allow coding against each outcome measure. The exclusion of one patient was pre-specified based on clinical grounds and is unlikely to have biased the results. 

Figure 4 below illustrates the significant improvement in discussions following implementation of the multistep pathway. This has not been fully reflected in the change in completed forms from baseline to post-intervention and was attributed by the team to multiple factors, including patient preferences to not complete the form, transfers to other wards, and those who did not meet eligibility criteria.

**Figure 4:**
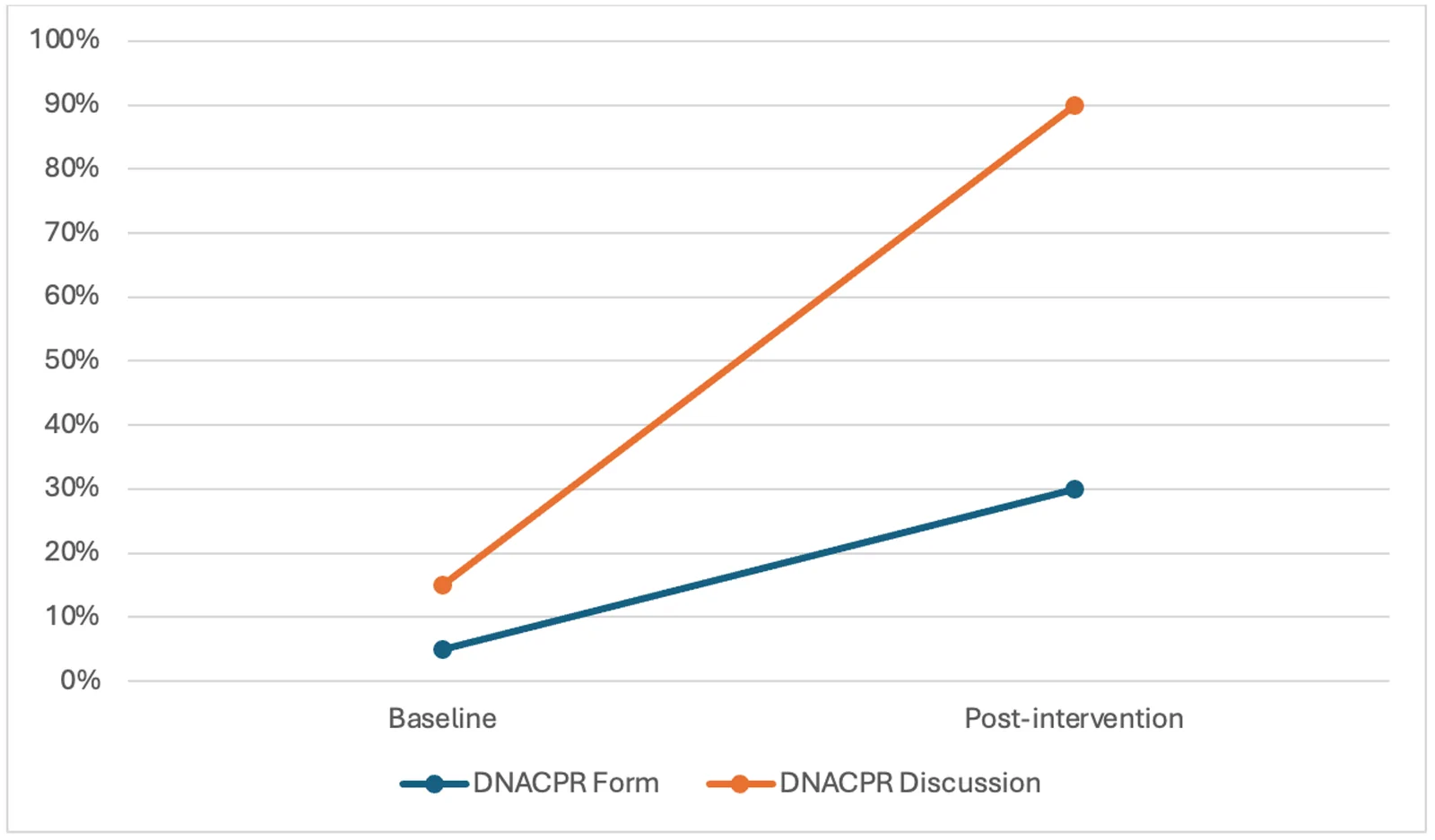
Pre- and post-intervention DNACPR outcomes DNACPR: do not attempt cardiopulmonary resuscitation

## Discussion

Early conversations with patients and family members or carers about frailty and DNACPR are a positive addition to the management plans, allowing for better treatment planning as well as improved patient outcomes. NHS England advises that DNACPR discussions form part of advanced care planning (ACP), which allows the patient a greater sense of involvement and the opportunity to reflect and share what matters most to them [14]. 

These findings are consistent with those of comparable QI work in older adult psychiatric settings nationally. Womersley et al. [11] reported marked improvements in DNACPR completion and online upload rates across two older-age psychiatry wards in NHS Fife following a similarly structured MDT-based intervention, including ward round prompts and peer education. The post-intervention DNACPR discussion rate of 90% compares favourably with baseline rates reported in the wider literature. A multisite audit of older-age psychiatric inpatient wards found that DNACPR status was discussed on admission in only 8% of cases and documented at the first MDT in only 10% [12], figures closely mirroring this project’s baseline of 15%. A contemporaneous audit across four old age psychiatry wards in Aneurin Bevan University Health Board identified particular deficits in documented discussion with patients themselves, with patient discussion present in fewer than half of DNACPR forms reviewed [13]. The emphasis on early patient and family engagement, supported by a dedicated information leaflet, directly addresses this gap and may account for the high discussion rate achieved in the re-evaluation cohort. 

The most important lesson from this project was the value of embedding quality improvement within existing ward structures. By anchoring frailty assessment and DNACPR planning to routinely occurring clinical events such as the MDT safety huddle and the Care Planning Meeting, the team was able to create sustainable change without requiring additional time, staffing, or infrastructure. Making the process explicit and expected, rather than left to individual clinician discretion, was the key driver of improvement. This is consistent with broader implementation science literature, which identifies the embedding of new behaviours into established clinical routines as a more effective and durable change mechanism than education-based interventions alone [15]. 

A secondary lesson concerned patient and family engagement. The development of the information leaflet proved important in facilitating DNACPR discussions, particularly with family members who had little prior knowledge of CPR and DNACPR. Several families required time to read and reflect on the information before they felt ready to discuss. This reinforced the importance of initiating the conversation early in the admission, rather than deferring it to the Care Planning Meeting alone. 

The MDT found that staging conversations with an initial frailty assessment, followed by a team discussion, followed by patient and family engagement over several contacts was more effective than attempting to complete DNACPR forms at a single meeting. This approach is consistent with national guidance and should be highlighted as a model for similar settings. 

This project has several limitations. The sample sizes were small (20 patients at baseline, 10 in the re-evaluation), limiting the statistical precision of the findings and rendering the project underpowered to detect smaller effect sizes, as reflected by the nonsignificant result for DNACPR form completion (p = 0.095). A one-month re-evaluation window may not fully capture the sustained impact of the intervention.

A significant structural limitation is the change in sampling frame between phases. The baseline cohort was a prevalence sample of all 20 patients currently admitted at a single point in time, while the re-evaluation cohort was an incidence sample of 10 consecutive new admissions over one month. These represent structurally different populations: long-stay patients with established care plans were over-represented at baseline, while the re-evaluation captured patients earlier in their admission trajectory. This difference may have influenced documentation rates independently of the intervention and represents a potential source of selection bias that the before-and-after design cannot fully account for.

A further important limitation is that the project's original dual aim included improving frailty assessment documentation to 100%, yet this outcome was not formally measured in the re-evaluation phase. This represents an incomplete evaluation of the stated objectives and is a more significant limitation than a simple omission, as it prevents any conclusion about whether the intervention achieved its frailty aim. This is acknowledged as the primary outcome for the next QI cycle, but the absence of re-measurement data means the full impact of the intervention cannot be assessed from this report alone.

The project used a before-and-after design, which, while practical for a service improvement context, does not allow attribution of change to specific intervention components, nor does it control for confounding factors such as staff turnover or seasonal variation in case mix. The absence of a run chart or statistical process control chart limits the ability to distinguish genuine sustained improvement from natural variation. Future cycles should incorporate these methods to strengthen the evidence of impact. No fidelity measures were included to assess how consistently each component of the pathway was delivered, and no balancing measures were collected to determine whether the intervention had any unintended negative effects on other aspects of care.

The 30% DNACPR form completion rate, while a considerable improvement from 5% at baseline, may appear modest. However, as the qualitative data in Table 7 demonstrate, the reasons for noncompletion were almost entirely clinical or patient preference-related rather than process failures. National guidance is explicit that a DNACPR form should only be completed where the clinical team, patient, and family have reached a shared understanding [9]. DNACPR form completion rate is therefore not an appropriate standalone measure of quality in this setting; the quality of the discussion and documentation of clinical reasoning are equally important. This article was written following guidance from Standards for QUality Improvement Reporting Excellence (SQUIRE) [16]. 

## Conclusions

This QIP has demonstrated short-term feasibility and a substantial improvement in DNACPR discussion documentation following implementation of a structured, MDT-driven, multicomponent pathway on a single older adult psychiatric inpatient ward. The before-and-after design supports an association between pathway implementation and improved documentation but cannot establish causality or identify which specific intervention component was responsible.

These results are consistent with published improvement work in older adult psychiatry and add to the growing evidence base for targeted QI interventions in this setting. The project addressed a clinically important and well-evidenced gap: older adults with SMI and significant frailty are at elevated risk of deterioration and death, and ensuring resuscitation decisions are made in a timely, patient-centred, and well-documented manner is both an ethical obligation and a key marker of quality of care.

The intervention is low cost, requires no additional staffing, and may be adaptable to other older adult psychiatric inpatient wards, though transferability should be evaluated in different contexts. The five-step pathway has been adopted as standard practice on The Oaks, with DNACPR status reviewed at each care planning meeting and at any point of clinical change; however, no formal follow-up audit data are yet available to confirm sustained improvement. Future cycles of this QIP will formally measure frailty assessment completion rates and will explore the use of a structured electronic prompt within records to further embed frailty scoring into the admissions workflow.

## Figures and Tables

**Figure 5:**
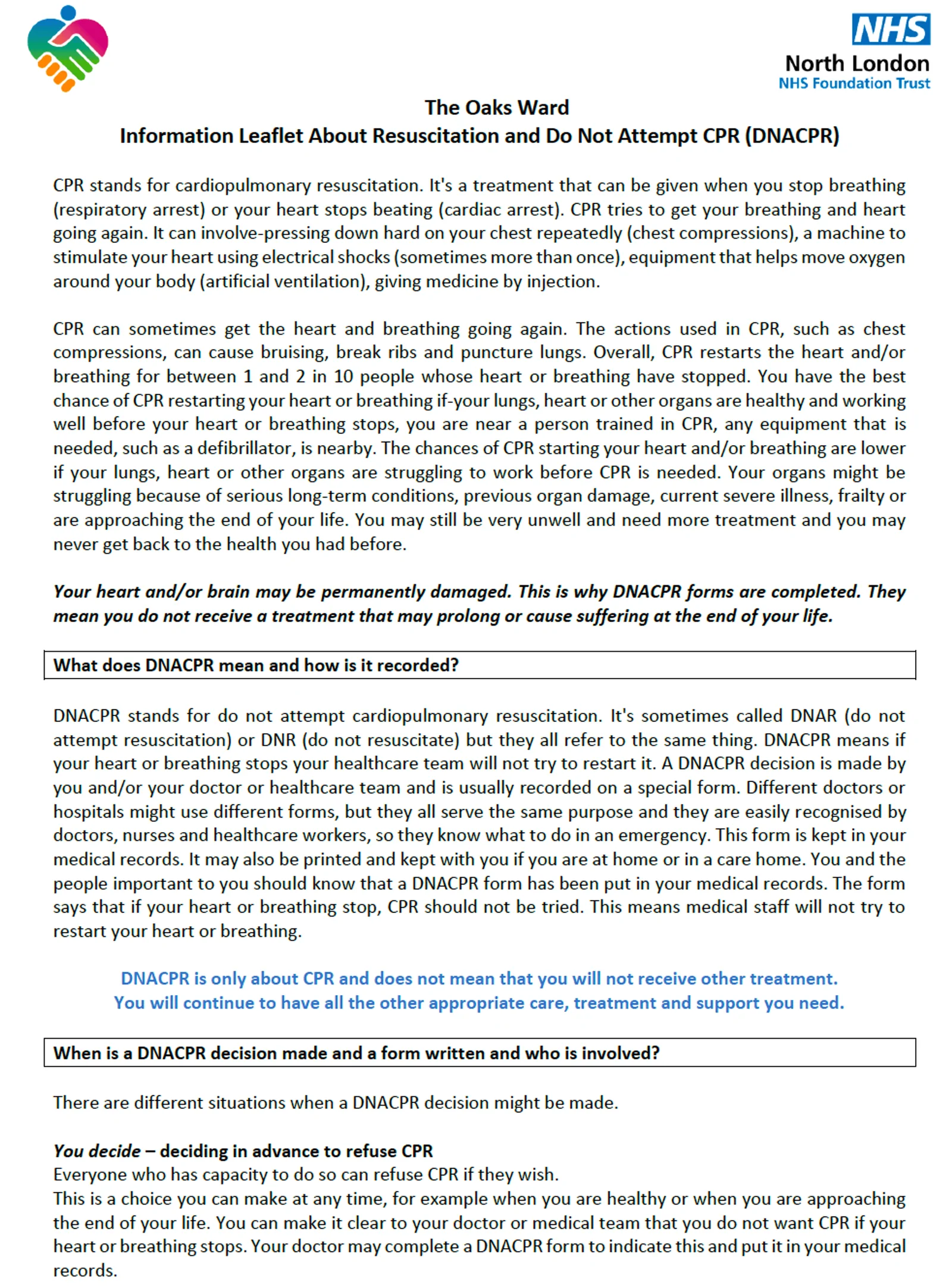
CPR and DNACPR leaflet, page 1 DNACPR: do not attempt cardiopulmonary resuscitation; CPR: cardiopulmonary resuscitation

**Figure 6:**
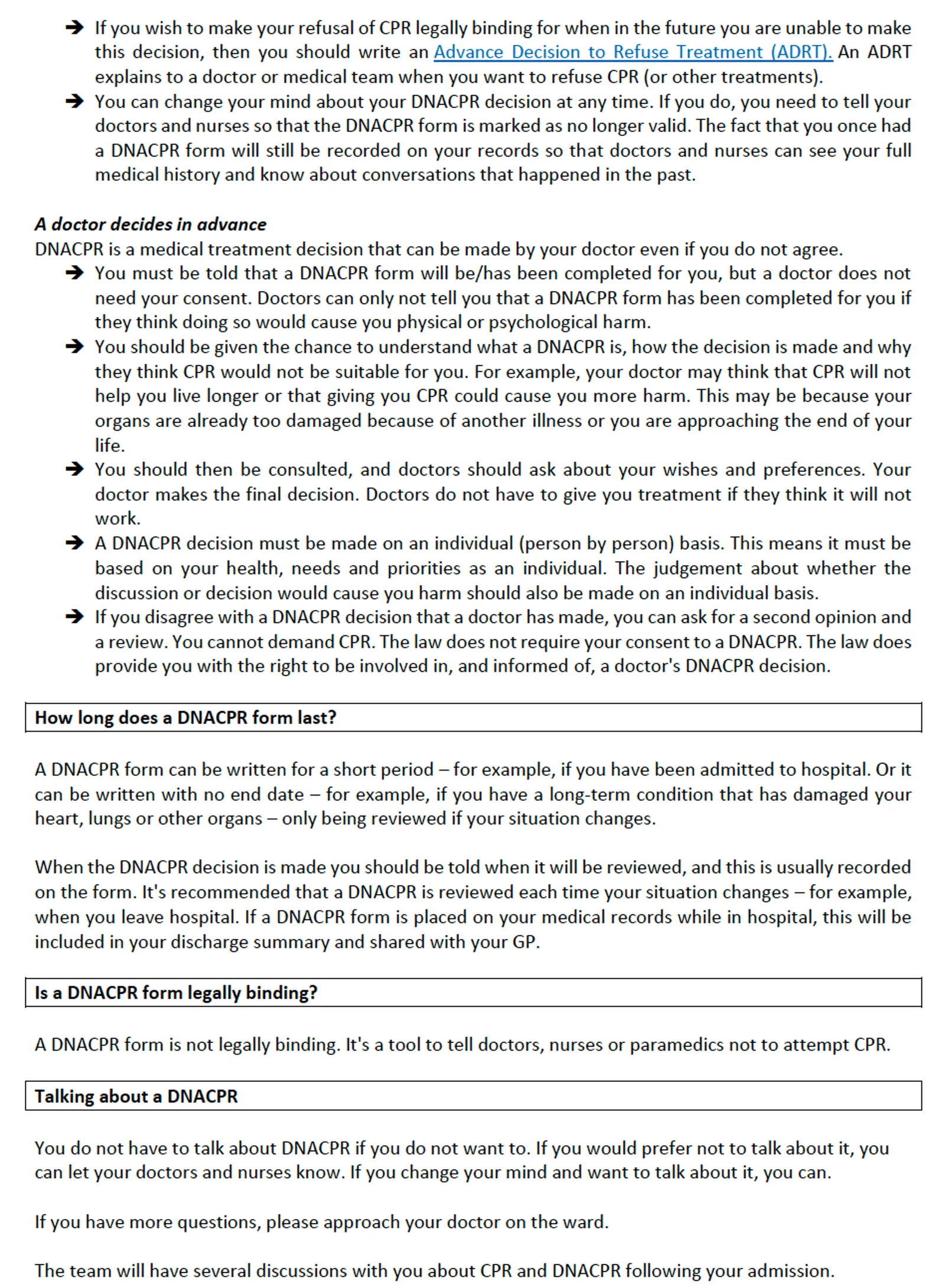
CPR and DNACPR leaflet, page 2 DNACPR: do not attempt cardiopulmonary resuscitation; CPR: cardiopulmonary resuscitation

## Appendices

**Table 6:** Baseline data DNACPR: do not attempt cardiopulmonary resuscitation

Patient	Frailty assessment completed?	Resuscitation discussion documented?	DNACPR form completed?
1	No	No	No
2	No	Yes	No
3	No	No	No
4	No	No	No
5	No	Yes	No
6	No	No	No
7	No	No	No
8	No	No	No
9	No	No	No
10	No	No	No
11	No	Yes	Yes
12	No	No	No
13	No	No	No
14	No	No	No
15	No	No	No
16	No	No	No
17	No	No	No
18	No	No	No
19	No	No	No
20	No	No	No

**Table 7:** Post-intervention data. Phase 4 (January-February 2025) DNACPR: do not attempt cardiopulmonary resuscitation

Patient	DNACPR discussion completed?	DNACPR form completed?	Additional comments
1	Yes	Yes	None
2	Yes	No	Patient and family chose active CPR
3	Yes	No	Patient and family chose active CPR
4	Yes	No	Patient transferred to another ward prior to completion of discussion
5	Yes	Yes	DNACPR completed after discussion with patient and husband
6	No	No	Minimal frailty. DNACPR discussion not felt appropriate
7	Yes	No	Patient admitted to medical ward for physical health concerns.
8	Yes	No	Following clinical improvement, further DNACPR discussions not felt appropriate
9	Yes	No	Further discussions not possible as patient transferred ward.
10	Yes	Yes	DNACPR completed after discussion with patient.
